# Characterization and phylogenetic analysis of the chloroplast genome of *Diospyros* aff. *oleifera*

**DOI:** 10.1080/23802359.2024.2419451

**Published:** 2024-10-28

**Authors:** Xingyu Zeng, Wenyan Zhao, Aihua Deng, Lixuan Xiang, Xuan Tang, Huan Li, Hanbin Yin, Rongjie Huang, Yulong Xiao, Yi Liu, Zui Yao, Yongle Liu, Zhitian Du, Kerui Huang

**Affiliations:** College of Life and Environmental Sciences, Hunan Provincial Key Laboratory for Molecular Immunity Technology of Aquatic Animal Diseases, Hunan University of Arts and Science, Changde, China

**Keywords:** *Diospyros* aff. *oleifera*, chloroplast genome, phylogenetic analysis, Ebenaceae

## Abstract

The *Diospyros* genus (Ebenaceae) has significant economic value. During field surveys, we discovered a *Diospyros* specimen showing morphological overlap with both *D*. *oleifera* and *D. kaki* var. silvestris, provisionally named *Diospyros* aff. *oleifera*. To resolve its taxonomy, we sequenced and analyzed its chloroplast genome. The complete chloroplast genome is 157,732 bp with a quadripartite structure. mVISTA analysis revealed unique sequence variations compared to related species. Phylogenetic analysis using 75 protein-coding genes grouped it with *D. oleifera*, indicating their close relationship. Our findings suggest this specimen likely represents a novel, undescribed species. This study provides insights into Diospyros diversity and a foundation for future research.

## Introduction

The genus *Diospyros* (Ebenaceae) encompasses over 500 species worldwide. *Diospyros* has a variety of uses, including edible fruits, precious timber, and ornamental purposes. Many plant parts of species within this genus are employed in various folk remedies, including treatments for bleeding, incontinence, insomnia, hiccups, and diarrhea (Rauf et al. 2017). The *Diospyros* genus plants are precious traditional medicinal resources in Traditional Chinese Medicine, Tibetan Medicine, and Ayurveda (Maridass et al. 2008).

Among these, *Diospyros oleifera* WCCheng 1935 (Shi 1935), a deciduous tree reaching up to 14 m in height, is characterized by gray to gray-brown pubescence covering most of its parts. Its distinctive oblong to oblong-lanceolate leaves, measuring 6.5–17 cm in length, typically exhibit 7–9 lateral veins. This diploid persimmon species serves as an excellent model for studying the genus due to its wide range of applications in biomedical science, food, timber, and chemical industries (Wu et al. 1996). Its rich content of antioxidants and other beneficial compounds contributes to its use in food production, biomedical research, and traditional medicine (Mai et al. 2023).

Another notable species, *Diospyros kaki* var. *silvestris* Makino 1908, is a deciduous tree distinguished by its densely yellow-brown pubescent young branches and petioles. Its leaves are comparatively smaller than those of cultivated persimmon trees, ranging from elliptic-ovate to oblong-ovate or obovate in shape. The fruit diameter of this variety does not exceed 5 cm (Makino 1908; Wu et al. 1996). *D. kaki* var. *silvestris* fruits are edible after de-astringency upon ripening and are traditionally used for various medicinal purposes, including the potential to prevent and treat high blood pressure and bleeding, slow down oxidation (Uchida et al. 1995), and maintain body temperature (Hibino et al. 2003). Furthermore, it can improve the functions of the lungs, stomach, and spleen (Direito et al. 2017). Leaf extracts have demonstrated effectiveness in the biosynthesis of palladium nanoparticles with significant antibacterial properties (Attar and Altikatoglu-Yapaoz 2018), and immature fruits can be used to extract persimmon lacquer (Renzi et al. 1997).

**Figure 1.**
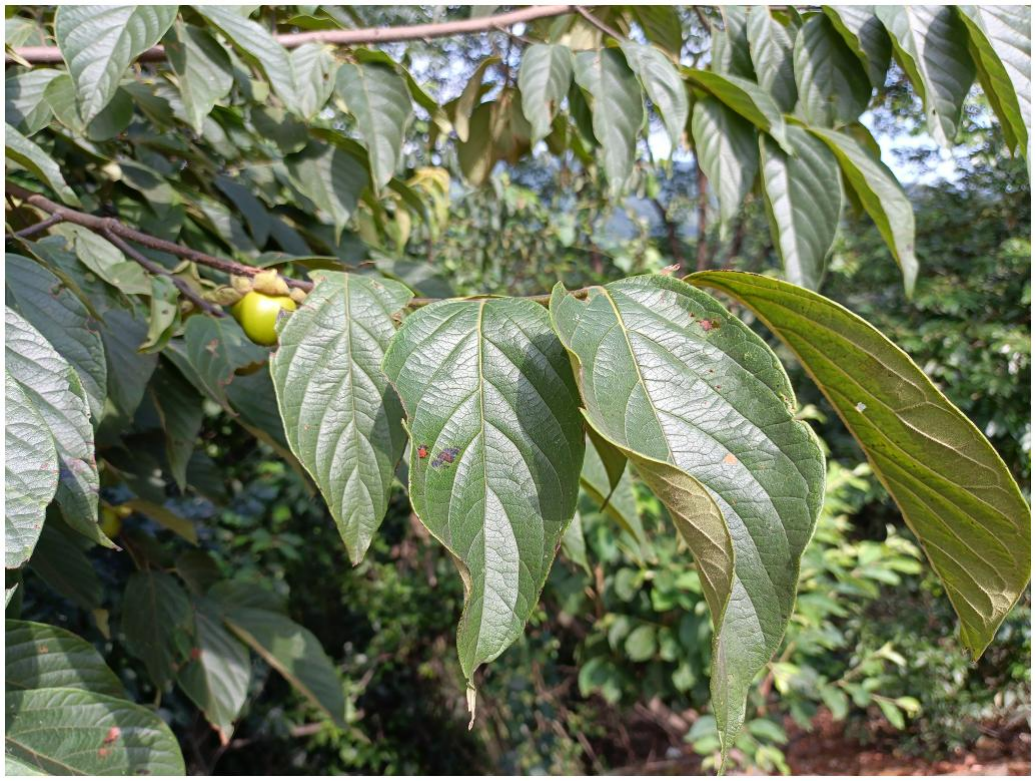
Picture of the collected sample of *Diospyros* aff. *oleifera*. The picture is self-taken by Hanbin Yin on 10 July 2022, the sample was collected on the same day from Nanyue District, Hengshan County and Eastern Hengyang County in Hunan Province, China (27.26474716°N, 112.71881232°E, altitude 360 m). The young branches and petioles of *Diospyros* aff. *oleifera*. are covered in soft yellow-brown hair, with variable elliptical leaves, pointed tips, and wide bases. The fruits have a diameter of no more than 5 cm.

During field surveys, we encountered a *Diospyros* specimen tentatively identified as *Diospyros* aff. *oleifera* (Figure 1). This specimen exhibits morphological characteristics that overlap with both *Diospyros oleifera* and *Diospyros kaki* var. *silvestris*. For instance, it shares the large leaves, oblong to oblong-lanceolate leaf shape, and 7–9 lateral veins characteristic of *D. oleifera.* However, it also possesses the densely pubescent petioles and leaf blades, and smaller fruit size (2–5 cm in diameter) more typical of *D. kaki* var. *silvestris*. This morphological ambiguity prompted us to investigate the chloroplast genome and phylogenetic placement of *Diospyros* aff. *oleifera* through chloroplast genome sequencing. Our aim is to determine its relationship to *Diospyros* oleifera and *Diospyros* kaki var. silvestris and lay a foundation for its further study in various areas.

## Materials

In terms of plant materials, the fresh fruits were harvested from wild persimmon trees on Heng Mountain in Nanyue District, Hengshan County, and eastern Hengyang County, Hunan Province, China (27.26474716°N, 112.71881232°E, altitude 360 m). The voucher specimen is preserved at the School of Life and Environmental Sciences, Hunan University of Arts and Science (Contact: Ke Rui Huang, huangkerui008@163.com, Voucher Number YS002).

## Methods

The total genomic DNA extraction and sequencing process followed the method of Zhang et al. (2023). First, we extracted total DNA from fruit samples stored in liquid nitrogen using the DNeasy Plant Tissue Extraction Kit (manufactured by Beijing Tiangen Biotech Co. Ltd., Beijing, China). Subsequently, we established a library and completed the sequencing on the Illumina HiSeq 2500 platform (provided by Personal Biotechnology Co., Ltd., Beijing, China). After using the fastq software to filter out low-quality reads, we excluded a total of 73,952,406 reads (Chen et al. 2018). Then, we performed de novo assembly of the chloroplast genome of *Diospyros* aff. *oleifera* using the GetOrganelle v1.7.5 (Jin et al. 2020) software and annotated the assembled genome with the CPGAVAS2 tool (Shi et al. 2019). Finally, we visualized the genome map using the CPGView (http://www.1kmpg.cn/cpgview/) tool.

To assess the sequence variation and identify potential regions of divergence among the chloroplast genomes of *Diospyros* aff. *oleifera*, *D. oleifera*, and *D. kaki* var. *silvestris*, we employed the mVISTA program (http://genome.lbl.gov/vista/mvista/submit.shtml). The complete chloroplast genome sequence of *D. oleifera* (NCBI accession number: NC070387) was used as the reference sequence. All available complete chloroplast genome sequences of *D. oleifera* and *D. kaki* were retrieved from the NCBI GenBank database and included in the analysis. The sequences were aligned using MAFFT v7.313 (Rozewicki et al. 2019) with default parameters.

For the phylogenetic analysis, we downloaded 40 chloroplast genomes from the GenBank database and set *Stewartia* as the outgroup and selected 75 common protein-coding genes from these genomes. We aligned each gene separately using MAFFT v7.313 (Rozewicki et al. 2019) and then masked the gene sequences using Gblocks 0.91b software (Guo et al. 2022). We concatenated the sequences of each gene to construct the supergene for each species. Under the TVM + F + I + G4 model with 5000 ultrafast bootstrap replications, we obtained the maximum-likelihood phylogenetic inference results using the IQ-TREE (Nguyen et al. 2015) software and the Shimodaira-Hasegawa-like approximate likelihood ratio test method.

## Results

**Figure 2.**
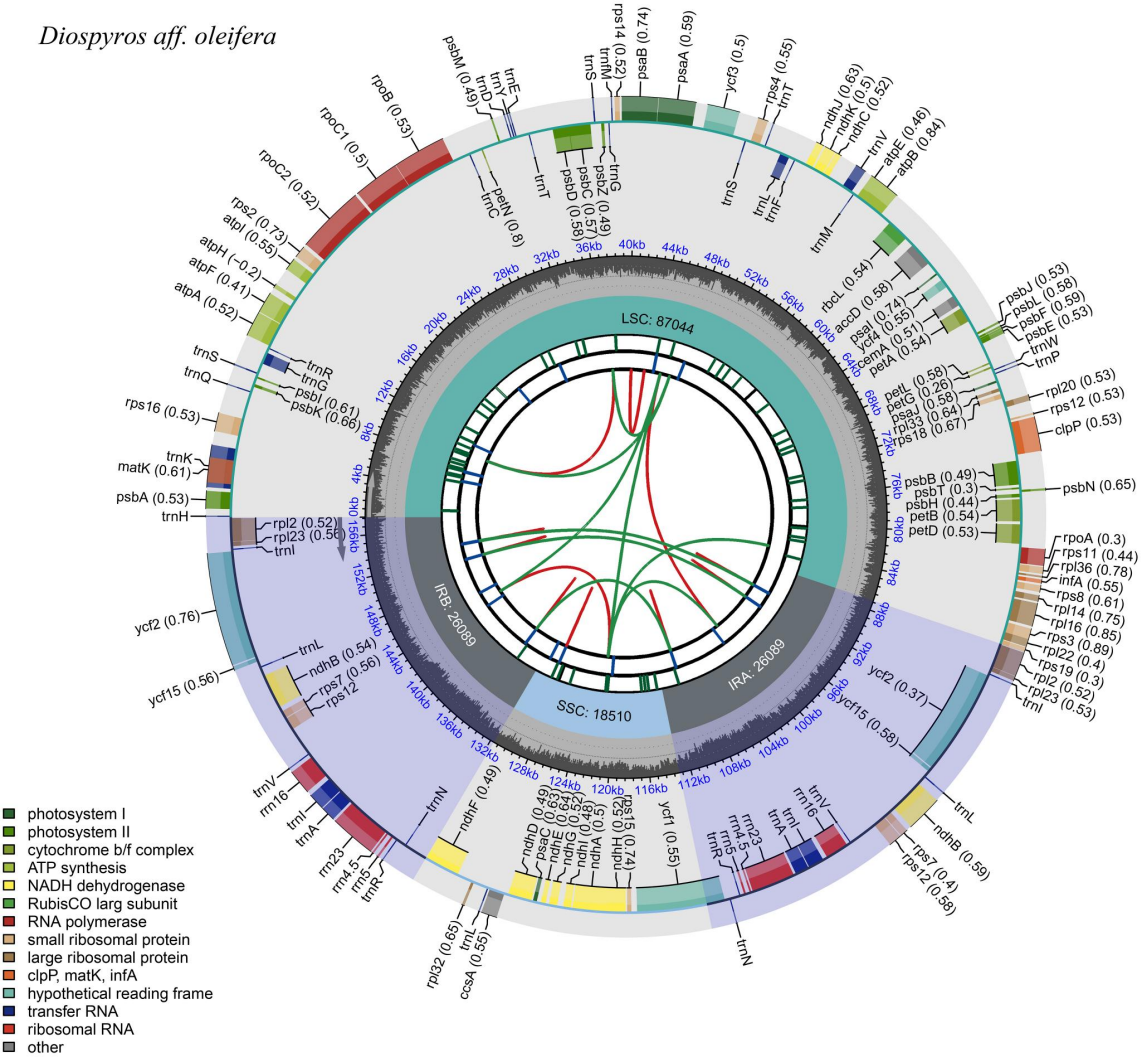
Gene map of the *Diospyros* aff. *oleifera*. chloroplast genome. From the center outward, the first track indicates the dispersed repeats. The second track shows the long tandem repeats as short blue bars. The third track shows the short tandem repeats or microsatellite sequences as short bars with different colors; the fourth track shows small single-copy (SSC), inverted repeat (Ira and Irb), and large single-copy (LSC) regions. The GC content along the genome is plotted on the fifth track; the genes are shown on the sixth track.

The chloroplast genome of *Diospyros* aff. *oleifera* is circular and 157,732 bp long, comprising a large single-copy region (LSC) of 87,044 bp, a small single-copy (SSC) region of 18,510 bp, and two identical reverse repeat (IR) regions, each 26,089 bp long, as illustrated in Figure 2 and Figure S1. The total G + C content of the chloroplast genome is 37.40%, with the G + C contents of the LSC, SSC, and IR regions being 35.41%, 30.81%, and 43.08%, respectively. The genome contains 132 genes, including 87 protein-coding genes, 37 transfer RNA genes, and eight ribosomal RNA genes. Among them, 18 genes are duplicated within the IR region. The chloroplast genome sequencing depth distribution is illustrated in Figure S2, and the structures of the cis- and trans-splicing genes are illustrated in Figure S3.

**Figure 3.**
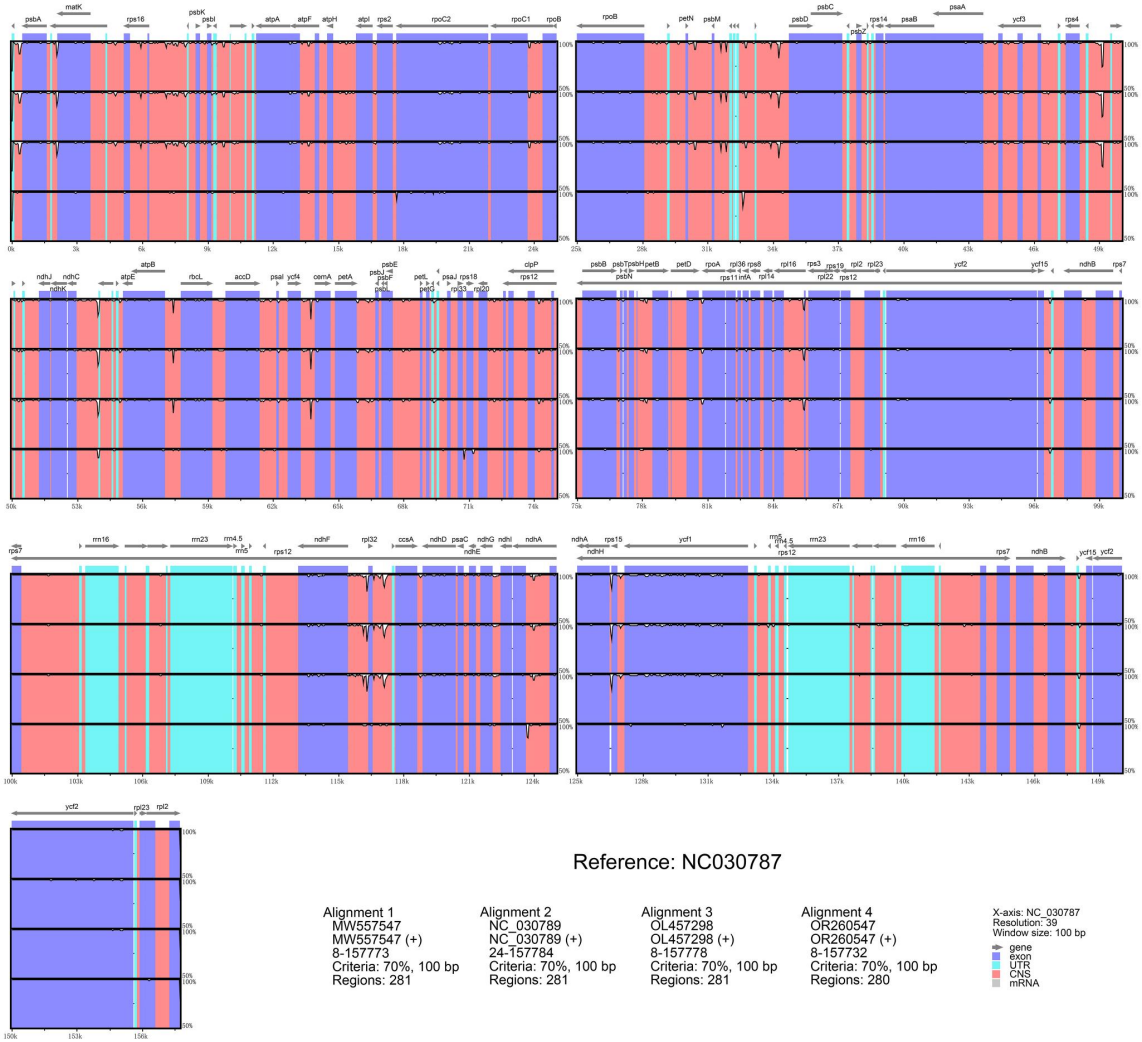
mVISTA-based chloroplast genome sequence comparison of *Diospyros* aff. *oleifera* with *D. oleifera* and *D. kaki*. This figure visualizes the chloroplast genome sequence variations among *Diospyros* aff. *oleifera*, *D. oleifera*, and *D. kaki* var. *silvestris* using mVISTA. The analysis utilized the *D. oleifera* sequence (NC070387) as a reference. Peaks above the horizontal axis indicate regions with high sequence conservation. The sequences included are NC070387 (*Diospyros oleifera*, reference sequence), OR260547 (*Diospyros* aff. *oleifera*), and MW557547, NC_030789, and OL457298 (*Diospyros kaki*).

To further investigate the relationship between *Diospyros* aff. *oleifera*, *Diospyros oleifera*, and *Diospyros kaki* var. *silvestris*, we conducted a chloroplast genome sequence variation analysis using mVISTA (Figure 3). We included all available *D. oleifera* and *D. kaki* (the parent species of *D. kaki* var. *silvestris*) sequences from NCBI and used the only available complete chloroplast genome sequence of *D. oleifera* (NCBI accession number NC070387) as a reference. The analysis revealed a high degree of overall similarity between the three species, suggesting a close phylogenetic relationship. However, detailed comparisons showed significant differences in the chloroplast genomes of *D.* aff. *oleifera* compared to both *D. oleifera* and *D. kaki*. Notably, *D.* aff. *oleifera* exhibited unique variations in specific regions when compared to *D. oleifera* – for instance, areas around 34 kb (near *psbD*), 57 kb (near *rbcL*), 64 kb (near *cemA*), 85 kb (within *rpl16*), and both within and adjacent to rpl32 (117 kb) showed greater differences. Variations were also observed at positions around 32–33 kb, 54 kb, 97 kb, and near ycf15 (148 kb), where *D.* aff. *oleifera* differed from *D. kaki*. Additionally, *D.* aff. *oleifera* displayed unique variations in regions such as near 18 kb (within *rpoC2*), 71 kb (near *rps18*), and within *ndhA* (124 kb), which were distinct from both species. Furthermore, the chloroplast genome length of *D.* aff. *oleifera* is 157,732 bp, which is 8 bp longer than that of *D. oleifera* (157,724 bp). Based on the mVISTA analysis, this length difference is attributed to the diverse genetic variations mentioned above, reflecting the influence of complex genetic factors. These variations, including insertions and deletions in specific regions, contribute to the overall genome length difference and underscore the unique genetic makeup of *D.* aff. *oleifera*. These findings strongly support the hypothesis that *Diospyros* aff. *oleifera* represents a potentially novel and undescribed species, distinct from *D. oleifera* and potentially *D. kaki* var. *silvestris*.

**Figure 4.**
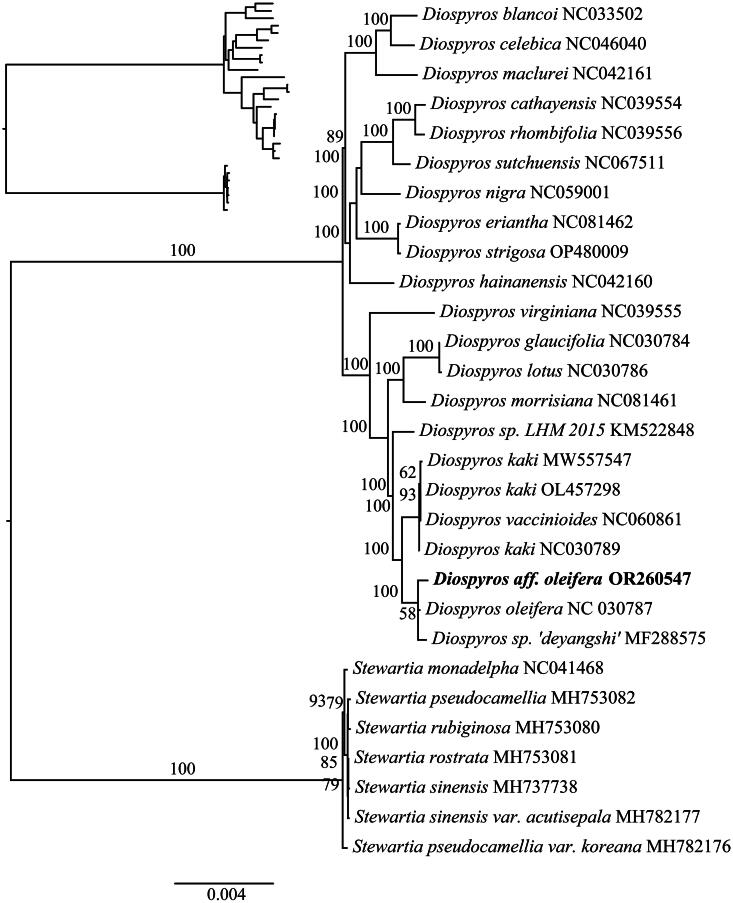
Maximum-likelihood tree of *Diospyros* aff. *oleifera* and 29 related species constructed by using the IQ-tree based on 75 protein-coding genes shared by all genomes. Bootstrap values are shown next to the nodes. The following sequences were used: *Diospyros blancoi* NC033502, *Diospyros celebica* NC046040, *Diospyros maclurei* NC042161 (Liu et al. 2018), *Diospyros cathayensis* NC039554 (Li et al. 2018), *Diospyros rhombifolia* NC039556 (Li et al. 2018), *Diospyros sutchuensis* NC067511 (Yang et al. 2024), *Diospyros nigra* NC059001, *Diospyros eriantha* NC081462, *Diospyros strigose* OP480009, *Diospyros hainanensis* NC042160 (Song et al. 2019), *Diospyros virginiana* NC039555 (Li et al. 2018), *Diospyros glaucifolia* NC030784 (Fu et al. 2016), *Diospyros lotus* NC030786 (Fu et al. 2016), *Diospyros morrisiana* NC081461, *Diospyros sp. LHM 2015* KM522848, *Diospyros kaki* MW557547 (Fu et al. 2016), *Diospyros kaki* OL457298, *Diospyros vaccinioides* NC060861, *Diospyros kaki* NC030789, *Diospyros* aff. *oleifera* OR260547, *Diospyros oleifera* NC030787 (Fu et al. 2016), *Diospyros sp. ‘deyangshi’* MF288575 (Li et al. 2018), *Stewartia monadelpha* NC041468 (Prince 2002), *Stewartia pseudocamellia* MH753082 (Prince 2002), *Stewartia rubiginosa* MH753080, *Stewartia rostrata* MH753081 (Prince 2002), *Stewartia sinensis* MH737738 (Prince 2002), *Stewartia sinensis* var. *acutisepala* MH782177, and *Stewartia pseudocamellia* var. *koreana* MH782176. *Stewartia* was set as the outgroup.

Figure 4 depicts a detailed phylogenetic tree constructed using the chloroplast genome of *Diospyros* aff. *oleifera*, which highlights its evolutionary relationship within the species. The study reveals that *Diospyros* aff. *oleifera* is closely related to *D. oleifera* and *D. sp. ‘deyangshi’*, belonging to the same evolutionary branch. Furthermore, *D. virginiana* and three *D. kaki* samples (MW557547, OL457298, and NC060861) are tightly clustered on one branch of the phylogenetic tree as a sister group of the clade of *Diospyros* aff. *oleifera*. This indicates that *Diospyros* aff. *oleifera* is more closely related to *D. oleifera* than to *D. kaki* or *D. kaki* var. *silvestris*, which, together with the mVISTA result, supports the hypothesis that *D.* aff. *oleifera* represents a potentially novel and undescribed species. Another notable thing is that *D. rhombifolia* and *D. nigra* are grouped on another faraway branch, in agreement with previous studies (Turner et al. 2013; Samuel et al. 2019). However, in this study, *D. celebica* is found to be closely related to *D. kaki*, *D. lotus*, *D. glaucifolia*, *D. virginiana*, *D. eriantha*, *D. nigra*, and *D. rhombifolia*, unlike previous research where *D. celebica* was not positioned near these species on the phylogenetic tree, representing a novel discovery (Turner et al. 2013; Samuel et al. 2019).

## Discussion and conclusions

This study presents the first comprehensive analysis of the chloroplast genome of a putative novel *Diospyros* species, tentatively identified as *Diospyros* aff. *oleifera*, discovered during field surveys. This specimen exhibits morphological characteristics that overlap with both *D. oleifera* and *D. kaki* var. *silvestris*, leading to ambiguity regarding its taxonomic classification.

Our chloroplast genome sequencing and comparative analysis provide compelling evidence to support the hypothesis that *Diospyros* aff. *oleifera* represents a distinct species. While the overall genome structure and gene content are similar to those of other *Diospyros* species, the mVISTA analysis revealed unique sequence variations in specific regions that differentiate *D.* aff. *oleifera* from both *D. oleifera* and potentially *D. kaki* var. *silvestris.* These variations suggest a unique evolutionary trajectory for this putative novel species.

The phylogenetic analysis further corroborates this conclusion. *Diospyros* aff. *oleifera* is placed within the same clade as *D. oleifera* and *D. sp*. ‘deyangshi’, indicating a closer relationship to *D. oleifera* than to *D. kaki* or *D. kaki* var. *silvestris*. This finding aligns with the observed morphological similarities between *Diospyros* aff. *oleifera* and *D. oleifera*, and finally supports the hypothesis that *D.* aff. *oleifera* is distinct from both *D. oleifera* and *D. kaki* var. *silvestris*.

The observed discrepancies in the phylogenetic placement of certain *Diospyros* species, such as *D. celebica*, compared to previous studies (Turner et al. 2013; Samuel et al. 2019) highlight the need for further investigation and more comprehensive sampling within the genus. The significant intraspecific variation observed within *D. kaki* samples also underscores the importance of considering intraspecific diversity when conducting phylogenetic analysis.

Our investigation of the chloroplast genome of *Diospyros* aff. *oleifera* reveals its unique genetic makeup and phylogenetic placement within the *Diospyros* genus. Comparative analysis and phylogenetic reconstruction strongly support its recognition as a potentially novel species, distinct from both *D. oleifera* and *D. kaki* var. *silvestris*. This discovery highlights the importance of continued exploration and genetic characterization of plant biodiversity. Further research, including nuclear genome analysis and more extensive morphological studies, is crucial to solidify its taxonomic status and explore its potential utility. This study provides a valuable foundation for future investigations into this intriguing *Diospyros* species.

## Supplementary Material

Supplemental Material.docx

## Data Availability

The complete chloroplast genome sequence of *Diospyros* aff. *oleifera* has been deposited in the GenBank database under the accession number OR260547. The associated BioProject, SRA, and Bio-Sample numbers are PRJNA1096847, SRR28573592, and SAMN40770032, respectively.
