# Supplementary material for: Characterization and phylogenetic analysis of the chloroplast genome of Diospyros aff. oleifera
Source: Mitochondrial DNA B Resour. 2024 Oct 28;9(11):1467–72. doi: 10.1080/23802359.2024.2419451 (PMC11520100; doi:10.1080/23802359.2024.2419451)
Supplement: Supplemental Material.docx [file TMDN_A_2419451_SM9087.docx]

**Supplemental Material**

**
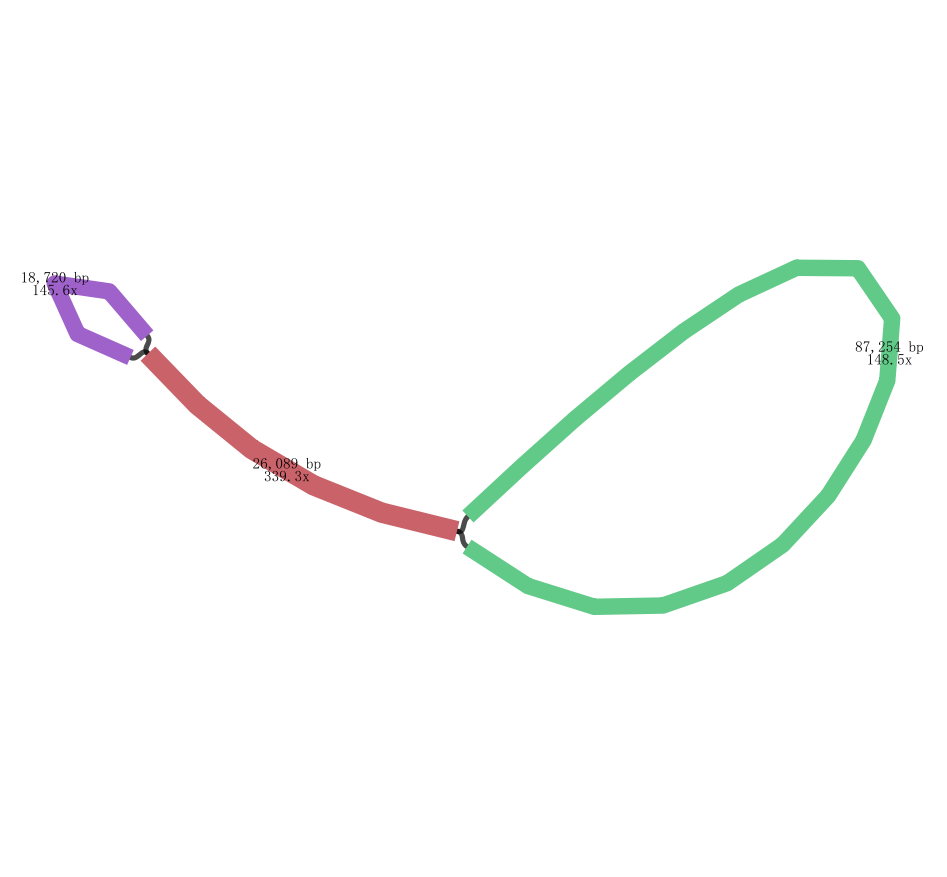
**

Figure S1. The schematic representation of the coverage depth for the entire chloroplast genome of *Diospyros aff. Oleifera* using Bandage. The numbers indicate the depths of different regions.


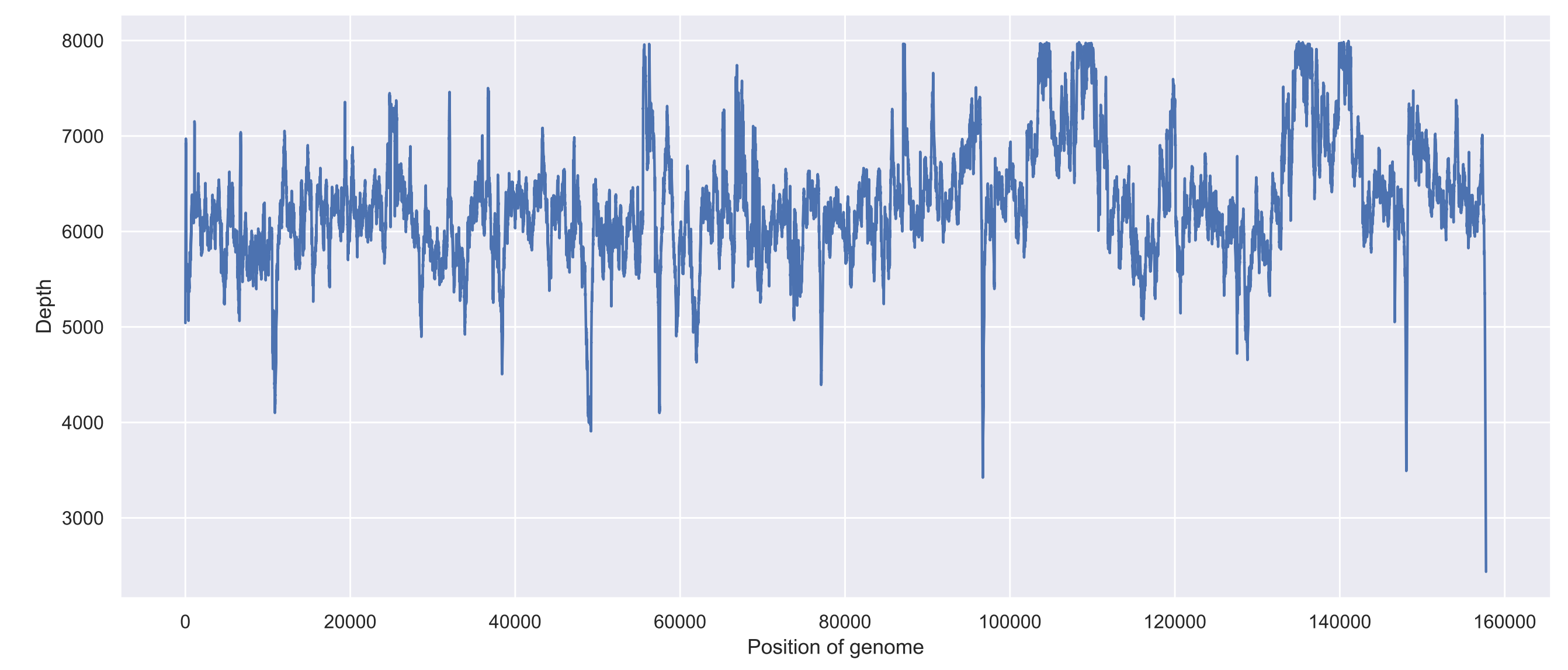


Figure S2. Chloroplast genome sequencing depth distribution for *Diospyros aff. Oleifera*. The graph illustrates the chloroplast genome sequencing depth distribution for species *Elaeocarpus duclouxii*, with the horizontal axis representing genomic position and the vertical axis indicating sequencing depth.

**
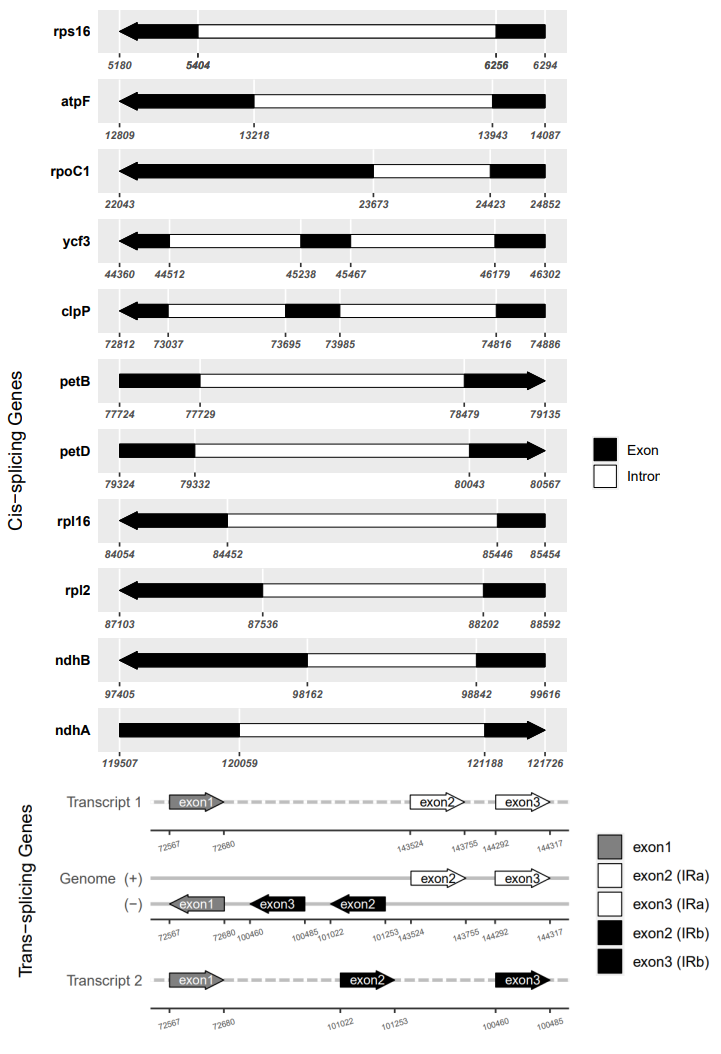
**

Figure S3. Schematic map of the cis-splicing genes and trans-splicing gene rps12 in the chloroplast genome of *Diospyros* aff. *Oleifera* using CPGView. The exons of the cis-splicing genes are shown in black; the introns are shown in white. The arrow indicates the sense direction of the gene. Please note that lengths of exons and introns are not drawn to scale.
